# Using Spatial Analysis to Predict Health Care Use at the Local Level: A Case Study of Type 2 Diabetes Medication Use and Its Association with Demographic Change and Socioeconomic Status

**DOI:** 10.1371/journal.pone.0072730

**Published:** 2013-08-30

**Authors:** Aletta Dijkstra, Fanny Janssen, Marinus De Bakker, Jens Bos, René Lub, Leo J. G. Van Wissen, Eelko Hak

**Affiliations:** 1 Unit of PharmacoEpidemiology & PharmacoEconomics (PE^2^), Department of Pharmacy, University of Groningen, Groningen, The Netherlands; 2 Population Research Centre, Faculty of Spatial Sciences, University of Groningen, Groningen, The Netherlands; 3 Groningen Center for Spatial Information, Faculty of Spatial Sciences, University of Groningen, Groningen, The Netherlands; 4 Netherlands Interdisciplinary Demographic Institute (NIDI), The Hague, The Netherlands; Bremen Institute of Preventive Research and Social Medicine, Germany

## Abstract

Local health status and health care use may be negatively influenced by low local socio-economic profile, population decline and population ageing. To support the need for targeted local health care, we explored spatial patterns of type 2 diabetes mellitus (T2DM) drug use at local level and determined its association with local demographic, socio-economic and access to care variables. We assessed spatial variability in these associations. We estimated the five-year prevalence of T2DM drug use (2005–2009) in persons aged 45 years and older at four-digit postal code level using the University of Groningen pharmacy database IADB.nl. Statistics Netherlands supplied data on potential predictor variables. We assessed spatial clustering, correlations and estimated a multiple linear regression model and a geographically weighted regression (GWR) model. Prevalence of T2DM medicine use ranged from 2.0% to 25.4%. The regression model included the extent of population ageing, proportion of social welfare/benefits, proportion of low incomes and proportion of pensioners, all significant positive predictors of local T2DM drug use. The GWR model demonstrated considerable spatial variability in the association between T2DM drug use and above predictors and was more accurate. The findings demonstrate the added value of spatial analysis in predicting health care use at local level.

## Introduction

In the near future, local health care use in European regions is expected to change as a consequence of demographic processes [1] as well as socio-economic change [2]–[4]. Firstly, population ageing increases the overall use of health care [2]. Secondly, regional socioeconomic polarization, which is strengthened by selective migration [3], causes growing regional inequalities in health [5]. Areas with population decline are expected to have a lower socioeconomic status, an older population and higher health care use at the population level [6].

On the other hand, the availability of services, amongst which is health care, is expected to decline in these regions [7]. These processes may result in a mismatch between supply and demand of health care [8], [9]. In order to deal with this mismatch, the provision of care needs to be carefully planned. By predicting local health care needs, research can help policy makers to effectively allocate health care. Spatial analysis targeting health care needs at local level can be vital in assessing which areas are at risk of becoming unhealthy and need more care in the future [10].

In this article, we study type 2 diabetes mellitus (T2DM) medication use as a case study for health care use at local level. This chronic disease is a major threat to health and has a high global burden [11]–[13]. Furthermore, prevention or early diagnosis of T2DM is expected to reduce health care costs and increase quality of life [14].

On the individual level, T2DM is associated with lower social economic status (SES) [3], [15]–[18]. Since regional population decline and ageing expectedly cause socioeconomic decline, we hypothesize that T2DM prevalence correlates negatively with SES and population growth and positively with population ageing. As the provision of health care may diminish in declining areas, we add variables pertaining to access to health care to our analysis. Furthermore, since SES displays clustering [19], it is likely that T2DM prevalence will display clustering as well. Finally, we hypothesize that there will be spatial variations in the relation between T2DM and the predictors at the local level; hence spatial analysis will have added value in this type of research.

Our objectives are (1) to find the associations between T2DM medication use at local level on one hand and socioeconomic and demographic factors and access to care factors on the other hand; (2) to develop a model that predicts T2DM medication use prevalence at local level and (3) to examine spatial variability in T2DM medication use, possible predictor and the relations between them. These objectives will help us to demonstrate the added value of spatial analysis in local health care use.

## Data and Methods

### Setting

We used the University of Groningen pharmacy database IADB.nl (for more information see: http://www.iadb.nl), an outpatient prescription database with approximately 500,000 persons registered at 55 pharmacies in the Northern Netherlands [20]. Its study population is representative for the Dutch population and has been used previously for diabetes research [21]. Each prescription record contains information on the prescription date; the quantity dispensed, the dose regimen, the number of days the prescription is valid, the prescribing physician and the Anatomical Therapeutic Chemical code (ATC code). Due to high pharmacy-commitment in the Netherlands, the medication records are expected to be virtually complete at the individual level, with the exception of over the counter drugs [22]. In accordance with the Dutch Law for the Protection of Personal Data (Wet Bescherming Persoonsgegevens) and the Declaration of Helsinki, patient data is not allowed to leave the pharmacy, therefore all patient data was depersonalized before entering the database. Each patient has a unique anonymous identifier. This study was approved by the IADB.nl advisory board.

### Study Area and Study Population

We analyzed aggregate data from 84 four-digit postal codes around the city of Groningen. In the IADB.nl each patient can be linked to a location down to the 4 digit postal code of the home address. However, because some individuals never receive any medication and others visit a pharmacy not covered in our database, the database coverage is not complete. To compensate this, an estimation of database coverage was made. In this estimation it was assumed that for municipalities in which all containing pharmacies are participating, the IADB has coverage of 100 percent. A population-ratio for each 1-year age and sex group is calculated for these municipalities. This is done by dividing the number of persons in the database by the total inhabitants in these areas (as recorded by Statistics Netherlands). In areas without complete coverage, the equivalent IADB coverage is estimated by multiplying the amount of persons in the database by the population-ratio.

In order to increase the validity of our analysis, we pruned 4-digit postal code areas by the following requirements: postal code areas need to have a database coverage > = 100 individuals or > = 25% of the total inhabitants and have at least two neighboring areas meeting the same criteria in order to be included in the research.

**Figure 1:**
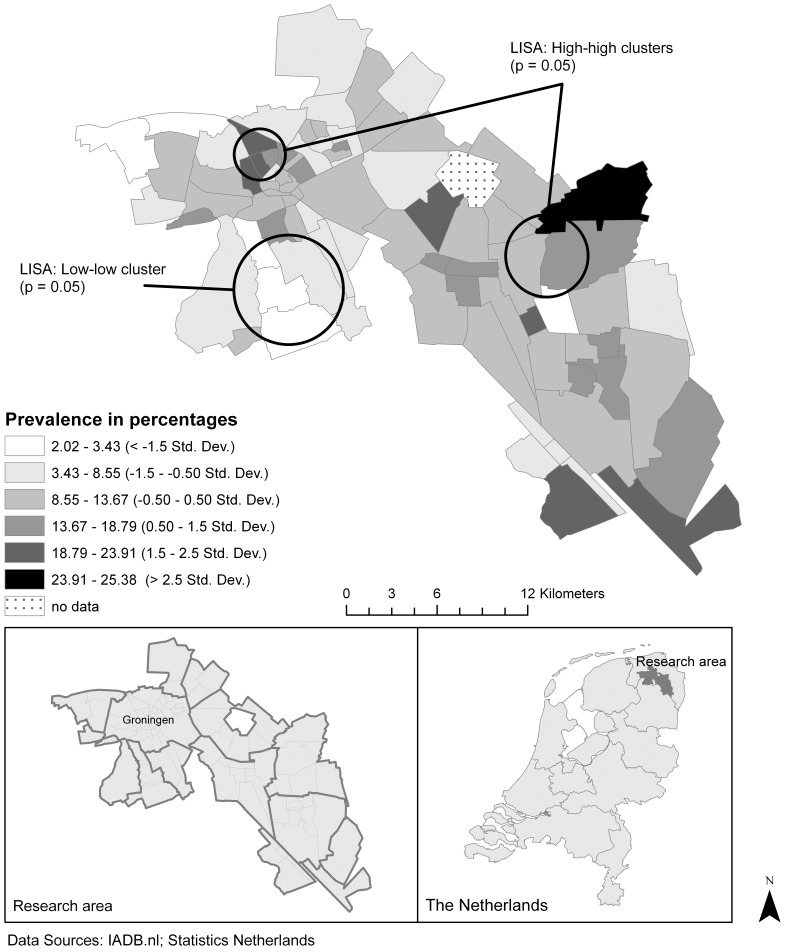
Five-year prevalence in the study area.

Our final study area encompassed 84 adjacent postal code areas situated around the city of Groningen (see Figure 1) and contained 23 pharmacies connected to IADB.nl. Based on data from Statistics Netherlands we estimate that this study area was inhabited by an average of 342,493 individuals during the research period, of whom 260,688 (76%) were in the IADB coverage. Amongst those, 98,753 were older than 45 and were used for calculation of our outcome measure.

### Outcome Measure

Our outcome measure was the five-year prevalence of type 2 diabetes mellitus (T2DM) medication use in persons 45 and older in the period of 2005–2009 by the 4-digit postal code area. Since the focus of this research is health care use, the definition of T2DM on the basis of medication use is the most relevant definition. Patients were considered to have T2DM if they received a prescription of any oral blood glucose lowering drug(s) (ATC-code starting with A10B). In the Netherlands these medications are rarely used off-label [23], therefore this definition is regarded accurate. To exclude patients with type I diabetes, who are generally younger and have different socio-demographic characteristics, we only included oral diabetic medication use in the outcome. Metformin is sometimes prescribed as fertility-medication. However in the Netherlands this is not standard practice and by targeting a population older than 45, we expect the group of patients using metformin for something other than diabetes to be low.

Prevalence at 4-digit postal code level was determined by dividing the total number of individuals older than 45 in 2007 receiving a prescription in the period of 2005–2009 by the average number of individuals older than 45 in 2007 in the estimated database coverage between 2005 and 2009.

### Predictors

Based on the hypotheses discussed in the introduction, we included 19 potential predictors, encompassing demographic variables, socio-economic variables and access to care variables. Data were obtained from the Statline database, made available by Statistics Netherlands [24].

To account for sex, the proportion of females in each area was included; to account for age, variables measuring the proportion of the population older than 60, respectively 80 and the proportion of pensioners were included; to account for population decline, a variable for population growth between 2005–2009 was computed; to account for the extent of population ageing, a variable for the difference in the proportion of persons over 65 (the legal pension age in the Netherlands at that time) between 2005 and 2009 was included. Furthermore, population density was calculated by dividing the number of inhabitants by the surface area of each postal code. To account for ethnicity, the proportion of nonwestern immigrants was included. Several variables pertaining to socioeconomic status were included: proportion of high income households; proportion of low income households; average income per person; average residential property value; proportion of the labor force that was on any form of social welfare or benefits; proportion of the households on social welfare and proportion of the households on disability benefits. Furthermore, we included some predictors pertaining to access to care: average distance to nearest GP; average amount of GP practices within three kilometers; average distance to the nearest hospital; average amount of hospitals within 20 kilometers. Distances are regarded as distances by road and averaged across all inhabitants in the area. An overview of variables and the years to which these apply is given in Table 1.

**Table 1:** Overview of variables.

Descriptive statistics and correlation with outcome measure										
Variable	Min.	Max.	Mean(Std. dev.)	Univariate Moran’s I (clustering)	P-value	Transformation	Pearson correlation with outcome measure	P-value	Bivariate Moran’s I (colocation) with outcome measure	P-value
**Outcome Measure**										
5-year prevalence T2DM 2005–2009 (% of populationaged 45 and over)	2.02	25.38	11.11 (5.15)	0.13**	0.02					
**Predictor Variables**										
Population growth (2005–2009) (% growth rate)	−86.7	300	3.41 (34.97)	−0.02	0.36		−0.26**	0.02	−0.04	0.36
Population ageing (2005–2009) (%point increase)	−14.98	10.71	1.01 (2.83)	0.1**	0.02		−0.2*	0.07	−0.06	0.26
Females (2007) (% of population)	40	54.68	49.68 (2.41)	0.21***	0.002		−0.1	0.91	−0.07	0.19
Persons aged 60 and over (2007) (% of population)	3.73	41.68	17.81 (7.82)	0.38***	0.001		0.05	0.67	−0.07	0.2
Persons aged 80 and over (2007) (% of population)	0	13.86	3.01 (2.71)	0.28***	0.001		0.1	0.36	−0.08	0.16
Nonwestern immigrants (2007) (% of population)	0	48	5.36 (6.82)	0.35***	0.001	Sq. root	0.4***	<0.001	0.11**	0.04
Social welfare/benefits (2007)(% of labor force)	7.66	28.86	14.92 (4.83)	0.18**	0.01		0.55***	<0.001	0.18**	0.01
Low incomes (2007) (% of labor force)	27.78	58.73	42.67 (7.81)	0.39***	0.001		0.5***	<0.001	0.22***	0.005
High incomes (2007) (% of labor force)	6.01	44.34	17.82 (8.10)	0.34***	0.001		−0.47***	<0.001	−0.23***	0.001
Average income per person (2007) (*1000 euros)	9.7	19.06	12.66 (1.88)	0.38***	0.001	log	−0.4***	<0.001	−0.20***	0.002
Average res. Property value (2007) (*1000 euros)	104.12	378.91	180.52 (57.33)	0.31***	0.001	log	−0.45***	<0.001	−0.23***	0.002
Pensioners (2007) (% of population)	3.43	36.13	16.24 (6.91)	0.45***	0.001	Sq. root	0.18	0.1	−0.05	0.33
Welfare (2007) (% of households)	0	20.93	4.54 (4.19)	0.31***	0.002		0.42***	<0.001	0.1*	0.06
Disability benefits (2007) (% of households)	3.71	25.36	11.22 (4.62)	0.49***	0.001		0.14	0.2	0.16**	0.01
Distance to nearest GP (2008) (kilometers by road)	0.24	6.83	1.60 (1.48)	0.48***	0.001		−0.04	0.7	0.02	0.32
Distance to nearest hospital (2008) (kilometersby road)	0.77	17.39	8.04 (5.14)	0.86***	0.001		0.06	0.59	0.15**	0.02
Amount of GPs (2008) (average number within3 km by road)	0	30.29	7.40 (8.96)	0.78***	0.001		0.14	0.22	0.09*	0.07
Amount of hospitals (2008) (average number within20km by road)	1	4	2.73 (0.7)	0.78***	0.001		−0.21*	0.05	−0.13**	0.04
Population Density (2005–2009 average) (averagenumber per 1km^2^)	1.63	1196.58	215.43 (342.85)	0.60***	0.001	log	0.08	0.45	0.03	0.31

*** significant at 1% confidence interval;

** significant at 5% confidence interval;

* significant at 10% confidence interval.

Predictors pertaining to SES, access to care and pensioners were only available at neighborhood level; the definition of neighborhood is: ‘part of a municipality with a homogenous socio-economic structure or planning (e.g. residential, industrial, recreational)’ [24]. Data for neighborhoods smaller than 50 persons is not made available publicly by Statistics Netherlands, as information pertaining to individuals might be distilled from this. To achieve compatibility with our outcome measure, predictors had to be estimated at 4-digit postal code level. For this we used the following procedure: missing cases of neighborhoods <50 individuals were replaced with district values (for proportions) or estimated using information of other neighborhoods in the district as well as district values (for absolute values). Neighborhood values were recalculated into postal code values using a proportional split method [25]. The proportional split method was done in ArcGIS 10.0; a self-written script was used to automate the process.

### Statistical Analysis

To explore statistical distributions in the outcome and predictor variables, we explored the range, mean and standard deviation. To ascertain the amount of clustering or dispersion of all variables, spatial autocorrelation was determined through global Moran’s I in OpenGeoDa 1.0 [26] using a row-standardized spatial weights matrix based on queen contiguity. The global Moran’s I is an indicator for spatial autocorrelation and measures whether the spatial pattern of a variable is clustered, dispersed or random [27]. The Moran’s I value ranges from −1 to 1, where −1 indicates perfect dispersion and 1 indicates perfect clustering. If the result of this test is statistically significant, the observed pattern displays either clustering or dispersion, if the result is not statistically significant, the spatial pattern is random. [27]. A Local Moran’s I (LISA) cluster map was created for T2DM medication use prevalence to determine local clustering. The LISA measures to what extent each area is surrounded by areas displaying the same features [27].

In the analytical phase, skewed variables were transformed exponentially or logarithmically to fit the linear assumption (see Table 1). We assessed non-spatial and spatial correlation. For our first research objective, we determined non-spatial correlation between T2DM prevalence and each of the predictors using Pearson correlation. To assess colocation between prevalence and each of the predictors, we calculated the bivariate Moran’s I for each predictor and prevalence, using OpenGeoDa 1.0. This measure of spatial correlation shows the association between prevalence at a given location and the predictor value in neighboring postal code areas [27].

Our second objective was aimed at finding a model that would best predict T2DM medication use prevalence at four digit postal code level, which meant that any combination of predictors was considered viable, as long as the model was valid. Multiple linear regression analysis was used to determine the association between the predictors and the outcome measure. We assessed which combination of predictors resulted in the best fitted valid model using the supplementary spatial statistics toolbox for ArcGIS 10.0 (available through ESRI) [28], while testing for normality, heteroskedasticity, multicollinearity and spatial dependence (using OpenGeoDa 1.0 for the latter) (see table 2). The residuals of the model were tested for spatial autocorrelation.

**Table 2:** Overview of regression results.

Outcome Measure: medication use prevalence over 45				
	*Multiple Linear Regression* (Adjusted R^2^∶ 0.43)			*GWR* (Adjusted R^2^∶ 0.45)
Variable	Coefficient	Std. Error	Probability	Range of coefficient
Intercept	−10.35***	3.50	0.004	−12.9 – −8.90
Population Ageing	0.48***	0.15	0.002	0.35–0.93
Social welfare/benefits	0.36***	0.11	0.002	0.14–0.42
Low incomes	0.24***	0.07	0.001	0.19–0.25
Pensioners	1.33**	0.52	0.013	0.84–3.34

*** significant at 1% confidence level;

** significant at 5% confidence level.

**Diagnostics Multiple linear regression:** F-statistics: 16.6 (p = 0); Koenker’s studentized Breusch-Pagan Statistic: 3.38 (p = 0.49); Jarque-Bera statistics: 4.17 (p = 0.12); Multicollinearity condition number: 21.8; Spatial autocorrelation of residuals: Moran’s I: 0.01 (p = 0.74); Langrange multiplier (lag): 0.82 (p = 0.36); Langrange multiplier (error): 0.08 (p = 0.78).

Finally, to address our third objective, we used Geographically Weighted Regression (GWR) to assess (1) to what extent the associations we found in the regular multiple linear regression model varied across the research area and (2) if taking this variation into account leads to more accurate prediction. The GWR measures the variation in the B-coefficients across the research area. A normal linear regression assumes that these correlations are the same for every area (spatially stationary), but a GWR assumes that relationships can be stronger in one area than in another (spatially non-stationary) [29]. The GWR was estimated using ArcGIS 10.0. To get an indication of the significance of the predictor coefficients in the GWR, the t-statistics were calculated by dividing the B-coefficients by their standard errors. Finally, the residuals of this model were tested for spatial autocorrelation as well. The GWR was compared to the multiple linear regression by comparing the sum of the squared residuals and the adjusted R-square.

## Results

Amongst the 98,753 individuals aged 45 and over, the five-year prevalence of type 2 diabetes mellitus (T2DM) medication use in the period of 2005–2009 was 13.2 percent. Throughout the different aggregate postal code areas, T2DM medication use prevalence ranged from 2.0 percent to 25.4 percent (Figure 1) with a mean of 11.1 percent and a standard deviation of 5.2 percent. T2DM medication use displayed a significant spatial pattern (Moran’s I = 0.13; p = 0.02) with a small amount of clustering. The LISA revealed two clusters with high prevalence and one cluster with low prevalence in and around the city of Groningen (see Figure 1 for locations).

Almost all predictors showed some degree of spatial clustering, the exception being population growth (Table 1). Predictors representing access to care demonstrated a high degree of clustering; these amenities are often located in central or urban places.

The predictors most strongly correlated with T2DM prevalence were the proportion of social welfare/benefits (r = 0.55), the proportions of low and high incomes (r = 0.50 and −0.47, respectively) and average residential property value (r = 0.45). Both population growth and the extent of population ageing were negatively correlated with prevalence. The variables for sex and age showed no statistically significant correlation.

Many of the predictors that were significantly correlated with T2DM medication use prevalence, also displayed significant colocation ( = spatial correlation) with prevalence (Table 1). However, spatial correlation was lower than non-spatial correlation, indicating that correlation is only partly determined by location. Although population ageing and population growth were correlated with T2DM medication use prevalence, there was no colocation as these variables displayed little spatial autocorrelation. Some predictor variables displayed colocation with T2DM medication use prevalence, although non-spatial correlation was absent. Most of these variables represented access to care.

The final linear prediction model (adjusted R^2^ = 0.43) included population ageing, social welfare/benefits, low incomes and pensioners, which all positively influenced local T2DM prevalence (Table 2). For population ageing a change in sign occurred compared to the Pearson correlation, indicating interaction between predictors. The residuals of this model showed no spatial autocorrelation.

**Figure 2:**
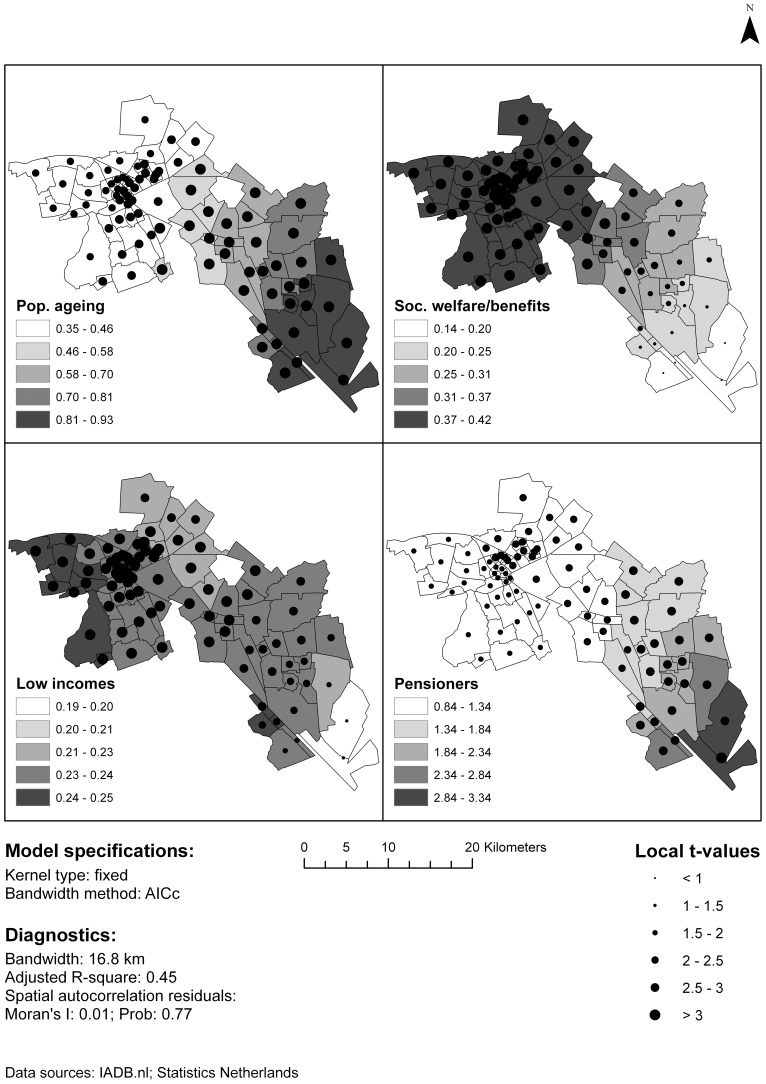
Results of Geographically Weighted Regression.

The GWR model (Figure 2, Table 2) showed that the local coefficients of most variables varied spatially. The coefficient for low incomes was fairly stable across the research area and within the confidence interval of the regular regression model, indicating a more or less constant relation between T2DM medication use prevalence and proportion of low incomes across the research area. Nonetheless the t-values indicate that results in the eastern part of the research area are less significant. The GWR coefficients of population ageing, social welfare/benefits and pensioners exceeded the confidence intervals found in the regular regression. The proportion of pensioners and population ageing was stronger associated with T2DM in rural areas than in and around the city of Groningen, whereas the proportion of social/welfare and benefits is less important (and significant) in rural areas, but more in urban areas.

The GWR model demonstrated higher discriminative value than the multiple regression model (adjusted R^2^ = 0.45 instead of 0.42), although not for all areas in the model as the local R^2^ ranged from 0.42 to 0.54. Also, the GWR displayed a better fit (sum of squared residuals  = 1064.95 instead of 1196.62). The residuals of the GWR displayed no spatial autocorrelation.

## Discussion

### Interpretation of the Findings

The objectives of this study were (1) to find the associations between T2DM medication use at local level on one hand and socioeconomic and demographic factors and access to care on the other hand; (2) to develop a model that predicts T2DM medication use prevalence at local level and (3) to examine spatial variability in T2DM, possible predictor and the relations between them.

With regards to the first objective, we found an association between SES and type 2 diabetes mellitus (T2DM) medication use at aggregate level. It seems that although age and sex are relevant when predicting T2DM at individual level (see for instance [30]), the differences are less pronounced at an aggregate level. Only the proportion of pensioners showed a relation with T2DM medication use at aggregate level.

Demographic change seemed less important as well. As hypothesized, we observed a negative correlation between population ageing and T2DM at the local level. However, the relation between population growth/decline and T2DM prevalence is weaker than we expected. A possible explanation for this is that house building is strongly regulated in the Netherlands and resulting population growth is caused more by planning than by natural processes at postal code level. A second explanation may be that our research area does not contain areas designated by local and national governments as declining regions for which policy actions are needed to cope with the effects of population decline [31]. Inclusion of these areas might lead to different outcomes.

The access to care variables show significant clustering, but little correlation with T2DM prevalence. An explanation for this could be the small range of the values of these variables, especially compared to the national scope [24]. Another explanation could be that no areas dealing with problems in health care distribution were included, as our selection was based on people using pharmacies, which is a form of care.

To meet our second objective, we strove to find the model that would best predict local T2DM medication use prevalence in our research area while making use of the available predictors. Our final model contained two variables relating to SES (proportion of local incomes and of social welfare and benefits), one to demographic composition (proportion of pensioners) and one to demographic change (extent of population ageing). Access to care variables seemed irrelevant in this multivariate analysis. This model did not include sex because (a) including this variable would cause multicollinearity as sex is highly correlated with pensioners (b) sex showed no relation with T2DM medication use prevalence in univariate analysis.

To meet our third research objective, variables were explored spatially and the prediction model was tested for spatial variability. We hypothesized that T2DM medication use prevalence would be clustered spatially and that this clustering would be similar in SES variables. Although many SES variables show a considerable amount of clustering, clustering of T2DM is limited. There is some colocation between T2DM prevalence and SES but the non-spatial correlation is higher.

The low amount of clustering in T2DM prevalence may be caused by the spatial randomness in population ageing, a predictor we also found to be associated with prevalence. This randomness might counterbalance any colocation T2DM prevalence may have with SES and also decrease the overall clustering of T2DM prevalence.

When the final prediction model was analyzed with GWR, spatial variability was found in the associations between T2DM and these predictors, but also in the significance of these associations. In general, the GWR model provides a more accurate prediction than the multiple linear regression model, thus proving its added value. As the relations in our model vary across space and as our predictors show specific spatial patterns, other Dutch areas may yield different models. This advocates using geographical methods when developing tools for intervention planning.

Our findings are consistent with other studies that found geographical variation in diabetes, although not always at a local scale level [32]. The study by Schlundt et al. [33] demonstrates that mapping and correlating diabetes and possible predictors is useful, as it helps researchers and policy makers find similarities in patterns. We found significant spatial clustering for prevalence and many of the predictors as well, particularly for variables representing access to care, that could be used to this end. Furthermore, the studies by Green et al. [34] and Bocquier et al. [35] demonstrate the use of regression of aggregated area data in predicting diabetes. Our findings regarding SES and diabetes were consistent with those in aforementioned studies. However, the fact that in our model the combination of SES, demographic composition and demographic change has a higher predictive power than merely SES variables proves that there is added value to be found in combining these different concepts when planning health interventions.

This research was done at a small scale level. Research on health care use at such a small scale level is particularly suited for identifying differences in health care use due to, for example, socioeconomic inequalities across areas [36]. Our findings suggest that such inequalities may indeed exist in the Netherlands, as the dimension that seems most important is the socioeconomic status. There are many ways in which socioeconomic status can relate to T2DM, often associated with lifestyle. For instance those with a lower SES may not have a healthy diet or an active lifestyle, as they can’t afford this or have little knowledge about these matters.

A related factor sometimes mentioned in literature is that those with a lower SES often have a diminished access to health care and that his may result in disparities in health across socioeconomic groups [37]. Given that the Netherlands has a universal health care system, in which every individual has basic insurance and GP practices are easily accessible [38], this effect should be minimalized in our study. Although we found very little relations between physical access to care and T2DM medication use, we still discovered a negative correlation between T2DM medication use and SES. This is consistent with studies done on T2DM in other populations with universal access to health care (see for instance [39], [40]) and it demonstrates that other processes are at work here, some of which may be targeted by interventions in certain socioeconomic groups [30], [33]. The localized approach we used in our research could be very helpful in such areas, as it looks at SES and health care use at a local level. Moreover, the situation we describe may be different in other areas in the Netherlands, since our GWR demonstrates that the relation between pensioners and diabetes varies greatly across space. These variations require for a more geographical approach in analysis to target areas at risk.

### Strengths and Limitations

This case study is an example on how to predict health care use at the local level, using spatial analysis. As health care needs to adapt to population decline, insights from such studies are very useful in planning future care. SES is something that expresses itself on this scale level rather than on higher levels, such as municipalities [41], therefore predictions based on local level data will be accurate and tailored to the needs of the population.

T2DM is very suitable as a case study as patients are easy to classify by medication use [16]. Moreover, this illness is amongst the illnesses that benefit from neighborhood based interventions [42]; insights from this research could aid the planning of such interventions.

Although the regular multiple linear regression provides a fairly accurate prediction model for the research area as a whole, the GWR is more useful as it illustrates that there is spatial variation in the associations between outcome measure and predictor variables. Furthermore in our study the GWR had a higher predictive power and better fit than the multiple linear regression. This showcases the added value of spatial analysis.

Another strength of this study is that it makes use of the IADB.nl database, which is valid and representative for the Dutch population, and very suitable for epidemiological research [20]. The data obtained from Statistics Netherlands is available nationwide and for different points in time and is considered to be very reliable for scientific research [24]. This makes our research easily extendable.

In the Netherlands, the average four digit-postal code size in 2007 was approximately 4000 inhabitants [24]. Since our average postal code size was 4077, our research area is very representative in this regard. Furthermore, the variability in our outcome measure T2DM medication use prevalence (2.0% to 25.4%) is considerable and we deem this to be another strength of this research. Statistics Netherlands previously measured the unstandardized one-year prevalence of T2DM to be 2.9 percent in 2005–2008 with a variation of 1.8–4.2 percent between different public health regions. Since postal codes are much smaller than public health regions (which generally encompass multiple municipalities [24]), it’s plausible that our variation was greater and our scale level should be more accurate in predicting local health care use [43]. The predictors may not be representative for the Dutch situation, as data from Statistics Netherlands reveals that our research area has a lower SES compared to the Dutch average and that the population lives further from health care than in most urban areas. A larger range of values within each predictor could produce different outcomes.

We believe that the possible bias in this study is limited. By applying strict selection criteria to select a study area, we tried to limit information bias in our outcome measure, however some of the samples may still be limited in representativeness as discussed in the previous paragraph.

Any study using statistics can be influenced by statistical uncertainty. In our case, not all variables displayed a linear distribution. We transformed some of the variables logarithmically or exponentially. However even after transformation, some distributions were not entirely normally distributed. This is why we have been mindful of diagnostics for non-normality and we have no reason to assume that non-normality is a problem in our regression models.

Secondly, a study using multiple predictor variables has to deal with correlation between the different variables. In our research we study many variables relating to SES and also several variables relating to age and access to care. The fact that some of these variables are interrelated stands beyond reason. For instance the proportion of low incomes is highly correlated with the proportion of high income. In this study we demonstrate that the concepts of SES and age composition can indeed be used to predict T2DM medication use. However, that does not mean all variables should be included when finding the best combination of variables. During our multivariate analysis we were very careful not to include variables displaying high multicollinearity. In order to assess this, we used two tools. Firstly, the exploratory regression tool in ArcGIS only selects models with low multicollinearity (variance inflation factor <7.5). Secondly, we assessed multicollinearity in our final model in OpenGeoDa (Multicollinearity condition number = 21; numbers below 30 are acceptable [27]).

Finally, this study is cross-sectional and consequently only measures association between the outcome measure and predictors which are not necessarily causal.

### Conclusions and Implications

This study displays the importance of SES and population ageing in predicting T2DM at the local level. In this research local regression is preferred over global regression as there is distinct spatial variability in the associations between T2DM and its predictors.

Analyzing and predicting medicine use at a local level can be very useful, as it helps locate areas at risk of becoming unhealthy [44]. SES characteristics on local level can reflect population differences in health status and are therefore very useful in predicting health care use and planning the allocation of care [45], [46]. Moreover, Potential T2DM patients could thus benefit greatly from lifestyle interventions, as discussed by Schlundt [36]. Our research demonstrate that the local scale level is ideal to target these patients, therefore policy makers should plan their interventions at the local level. Areas in which intervention is most profitable may be found by studying local SES, age composition and population ageing.

Future research can benefit from a localized approach when explaining or predicting local health care use. In this study we took spatial analysis a step further than other studies on type 2 diabetes that used a geographical approach, by examining colocation besides regular correlation and by estimating a GWR model. Moreover, we added variables for demographic change. Both of these aspects have proven to be useful in this study and could be applied to other epidemiological studies as well. Using spatial analysis in this type of research is very useful, as it reveals spatial variation in the relations between health care use and the different predictors, making the prediction more accurate and tailored to neighborhoods.
